# Performance Comparison of SOI-Based Temperature Sensors for Room-Temperature Terahertz Antenna-Coupled Bolometers: MOSFET, PN Junction Diode and Resistor

**DOI:** 10.3390/mi11080718

**Published:** 2020-07-24

**Authors:** Durgadevi Elamaran, Yuya Suzuki, Hiroaki Satoh, Amit Banerjee, Norihisa Hiromoto, Hiroshi Inokawa

**Affiliations:** 1 Graduate School of Science and Technology, Shizuoka University, Hamamatsu 432-8011, Japan; durgaelamaran@gmail.com; 2 Faculty of Engineering, Shizuoka University, Hamamatsu 432-8561, Japan; suzuki_yuya@fujidenki.co.jp; 3 Research Institute of Electronics, Shizuoka University, Hamamatsu 432-8011, Japan; satoh.hiroaki@shizuoka.ac.jp; 4 Physics Department, Bidhan Chandra College, Asansol 713 303, India; amitbanerjeeiacseru@gmail.com; 5 Graduate School of Integrated Science and Technology, Shizuoka University, Hamamatsu 432-8561, Japan; hiromoto.norihisa@shizuoka.ac.jp

**Keywords:** antenna-coupled bolometer, silicon-on-insulator (SOI), temperature sensor, responsivity (*R_v_*), noise equivalent power (NEP), thermal response time (*τ*)

## Abstract

Assuming that the 0.6-μm silicon-on-insulator (SOI) complementary metal–oxide–semiconductor (CMOS) technology, different Si-based temperature sensors such as metal-oxide-semiconductor field-effect transistor (MOSFET) (n-channel and p-channel), pn-junction diode (with p-body doping and without doping), and resistors (n^+^ or p^+^ single crystalline Si and n^+^ polycrystalline Si) were designed and characterized for its possible use in 1-THz antenna-coupled bolometers. The use of a half-wave dipole antenna connected to the heater end was assumed, which limited the integrated temperature sensor/heater area to be 15 × 15 µm. Our main focus was to evaluate the performances of the temperature sensor/heater part, and the optical coupling between the incident light and heater via an antenna was not included in the evaluation. The electrothermal feedback (ETF) effect due to the bias current was considered in the performance estimation. A comparative analysis of various SOI bolometers revealed the largest responsivity (*R_v_*) of 5.16 kV/W for the n-channel MOSFET bolometer although the negative ETF in MOSFET reduced the *R_v_*. The noise measurement of the n-channel MOSFET showed the NEP of 245 pW/Hz^1/2^, which was more than one order of magnitude smaller than that of the n^+^ polycrystalline Si resistive bolometer (6.59 nW/Hz^1/2^). The present result suggests that the n-channel MOSFET can be a promising detector for THz applications.

## 1. Introduction

Recently, the terahertz (THz) wave with frequencies from 0.3 to 3 THz have shown exciting possibilities in the area of non-invasive inspection and imaging for their transparency to non- polarized materials and spectral fingerprints of organic- and bio-macromolecules [1]. Compared with X-rays and millimeter waves, THz waves are non-ionizing and have a high spatial resolution. Hence, they can be effectively used for imaging concealed objects, material identification, and biomedical diagnosis [2]. Such a wide scope of the THz technology highly necessitates the development of low-cost, high sensitive, room-temperature THz detectors. Photonic detectors operated in the THz range are inevitably affected by the small energy of the photon resulting in an additional cryogenic cooling system [3]. The recently developed semiconductor based quantum well photodetector [4], antenna-coupled silicon dot photodetector [5], and patch antenna-coupled quantum well photodetector [6] for THz detection shows a noise equivalent power (NEP) less than 1 pW/Hz^1/2^ at low temperatures. Cooling requirements of photon detectors are the main obstacle for extensive usage. The possible choices of room-temperature THz detectors are microelectromechanical system (MEMS)-based resonators, electronic detectors or receivers such as the Schottky barrier diode (SBD), FET, etc., and thermal detectors such as the bolometer. Recently, GaAs based MEMS thermistors utilizing the thermo-mechanical transduction scheme were fabricated for the detection of THz radiation. The temperature change in the MEMS resonator was observed as a resonance frequency shift and realized the NEP of about 90 pW/Hz^1/2^ and the operation bandwidth of several kHz simultaneously [7]. In SBD, the electric field of the incoming radiation received by an antenna is rectified by the difference between the forward and reverse flow of the current. This in turn generates a dc signal for the corresponding input power. In the case of FET, the detection of radiation results from the rectified alternating current (AC) induced by the transistor channel’s nonlinear properties. The photo response that appears between the source and drain is in the form of a direct current/voltage and is proportional to the radiation power. SBD has been used for room-temperature THz detection [8], and its evolution in planar design has widened the usage [9]. Furthermore, the integration with CMOS technology realized the arrayed detectors for imaging [10]. Recently, the distributed self-mixing in the FET channel emerged as a mechanism of rectification at frequencies above its cutoff frequency [11,12,13], and the implementation in the CMOS platform has been studied intensively [14,15,16,17,18]. However, the performance of SBD and FET degrades rapidly with frequency [13], and thermal detectors, whose performance is relatively independent of frequency, become attractive at higher frequency if the temperature sensing scheme and thermal isolation structure are properly improved to attain higher performance [19]. The phenomenal thermal detector changes its material properties as a result of the temperature rise caused by the input radiation [20,21]. For a longer wavelength (*λ*) such as in the THz regions, due to the diffraction limits in focusing, the size of the absorber should be large in proportion to λ [20]. The realization of a large absorber and sensor with reduced thermal conductance and low heat capacity is quite difficult. To address this issue, the antenna-coupled bolometer is introduced to detect THz radiation at low frequencies. The antenna-coupled microbolometer has been extensively studied from the past three decades [22], in which the radiation signal is received by an antenna and converted to heat by the heater.

The temperature sensor is the most important element of the bolometer, and many varieties of sensors are available, such as resistor (thermistor), pn-junction diode, FET, thermocouple, etc. A wide variety of thermistor (resistor) materials such as vanadium oxide (*VO_x_*) [23,24,25,26], metal [27,28,29], and semiconductors [30,31,32,33] were used in conventional absorber type bolometers. For this kind of temperature sensor, the temperature coefficient of resistance is a vital parameter. Amorphous silicon (a-Si) [33] and *VO_x_* are the most commonly used temperature sensing materials, since they have a high temperature coefficient of resistance (TCR). However, a-Si based temperature sensor requires process optimization when integrated with a read-out circuit due to its sensitivity to the processing conditions during and after the deposition, and *VO_x_* is not a standard material for CMOS integration, though it has a moderate TCR and resistivity. Other metals such as titanium and platinum are realized as a temperature sensor, since it has a low thermal conductivity and resistant to oxidation, respectively. On the other hand, the single crystal or polycrystalline Si has been studied extensively since it is compatible with CMOS integration. Recently, the pn-junction diode based thermal sensor has been investigated for infrared detection [34,35,36]. Similarly, thermocouple detectors [37] have been investigated to sense infrared radiation, the performance mainly depends on the Seebeck coefficient of the temperature sensing material. Metal-oxide-semiconductor field-effect transistor (MOSFET) bolometers, in which the threshold voltage shift by the temperature is utilized as a temperature sensing scheme, and the amplification function of the MOSFET results in a large output, i.e., a large responsivity (*R_v_*), have also been studied [19,38,39].

In the current work, our main focus is to compare the performance of various SOI-based temperature sensors (n/p-channel MOSFET, undoped/p^-^ body doped pn-junction diode, n^+^/p^+^ single crystalline silicon, and n^+^ polycrystalline silicon resistor) in a 1-THz antenna-coupled bolometer design [40]. Thermal detectors, including bolometers, are fundamentally limited by the temperature fluctuation noise [20,41] as will be discussed later, and NEP in the order of 100 pW/Hz^1/2^ is desirable for active imaging applications [42]. Furthermore, a high responsivity to THz radiation is beneficial since it relaxes the requirement of a low-noise amplifier in the readout circuit. In the current work, the figure of merits including *R_v_*, NEP, and τ were evaluated and compared. To the best of our knowledge, this is the first report to make a comparison among different sensors under the common constraints of the SOI CMOS design rule.

## 2. Structure of Antenna-Coupled Bolometers

The main subject of this research is to evaluate and compare various temperature sensors in the 1-THz antenna-coupled bolometer design [40], where a half-wave gold dipole antenna is assumed. The common heater design is assumed with its both ends connected to the antenna rods. In such a design, the sensor/heater is placed at the center, and the length of this part should be sufficiently smaller than the total length of the antenna, i.e., 50 µm, for 1 THz. Due to the presence of the substrate with a high dielectric constant, the antenna length is shortened from the half wavelength in the free space. In the assumed design, an antenna with a width-length ratio of 0.1 is placed on a silicon substrate with the dielectric constant of 11.67, the optimum heater resistance is found to be 40 Ω [43], whereas the resistance of the gold antenna rod limited by the skin effect is 0.99 Ω at 1 THz and almost negligible. However, in the present work, the available resistance 836 Ω of the polysilicon heater is away from the optimum value, resulting in one third of the optimum absorption efficiency, and therefore only the heater/sensor component has been characterized for partial optimization.

**Figure 1:**
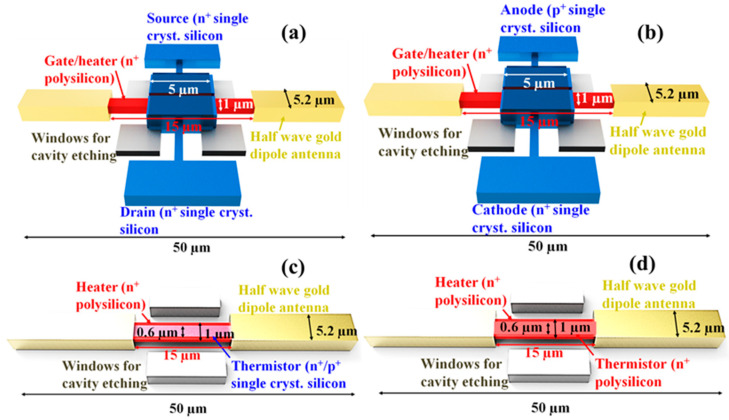
The structure of assumed antenna-coupled bolometers and its dimensions. (**a**) P or n-channel MOSFET bolometer, (**b**) pn-junction diode bolometer, (**c**) p^+^ or n^+^ single crystalline silicon resistive bolometer, and (**d**) n^+^ polycrystalline silicon resistive bolometer.

Bolometers without an antenna of the present work were fabricated by the standard Si CMOS technology instead of the complex non-standard lift-off process used in our previously reported works [44,45]. The key advantages of the Si CMOS technology are good reproducibility and universalities of the results. For the purpose of evaluating the temperature sensor in an antenna-coupled design, the novel high performance antenna-coupled metallic microbolometer structure on a high-resistivity Si substrate for sensing at around 1 THz was adopted. This limits an integrated sensor/heater area to be 15 × 15 µm. The minimum feature size of 0.6 µm is used in the present CMOS fabrication technology. Based on the measurement results of the fabricated device, the performance of the assumed device was estimated under the constraints of the antenna-coupled design at 1 THz and the 0.6-μm design rule. The structures of the assumed devices with its dimensions are shown in Figure 1, where a half-wave gold dipole antenna is directly coupled at both ends of the heater. The dimension of polycrystalline heater is commonly set for all the bolometers. An insulation layer stacked between an integrated sensor/heater is located at the center of an antenna, and it is suspended above the cavity in the Si substrate. The temperature sensor is electrically separated, but thermally connected with the heater. Figure 1a,b represents the assumed design of n/p-channel MOSFET and undoped/p^-^ body doped pn-junction diode bolometers, respectively. In MOSFET and diode bolometers, an additional lead with the length of 5 µm is connected on each side of its gate electrode, and it is extended towards the antenna. The gate electrode together with the lead serves as a heater. The thickness of the gate oxide, gate polysilicon, SOI, and buried oxide (BOX) are 7, 130, 100, and 200 nm, respectively. Figure 1c,d demonstrates the assumed structure of resistive bolometers with a n^+^/p^+^ single crystalline silicon thermistor and n^+^ polysilicon thermistor, respectively.

Table 1 represents the dimensions of the fabricated and assumed device structure. The anode/cathode width of the diode is assumed to be 5 µm. Similarly, the heater is assumed for the resistor with the width of 1 µm and length of 15 µm. Polycrystalline silicon is intended to be used as a heater material for all the bolometers. The single crystalline silicon was used to fabricate the source/drain and anode/cathode of FET and pn-junction diode, respectively. Three different kinds of Si materials such as n^+^/p^+^ single-crystal Si and n^+^ polycrystalline Si were used to fabricate the temperature sensing part of the resistive bolometer.

## 3. Estimation Procedure of Performance Metrics

Performance metrics such as *R_v_,* NEP, and *τ* of the bolometers are evaluated from the electrical response and noise measurements. The constant-current (CC) load (*I_b_*) of 10 µA was connected to all the devices for a fair comparison among them. This section explains the procedure used to estimate various performance metrics.

### 3.1. Estimation Procedure of Rv

**Figure 2:**
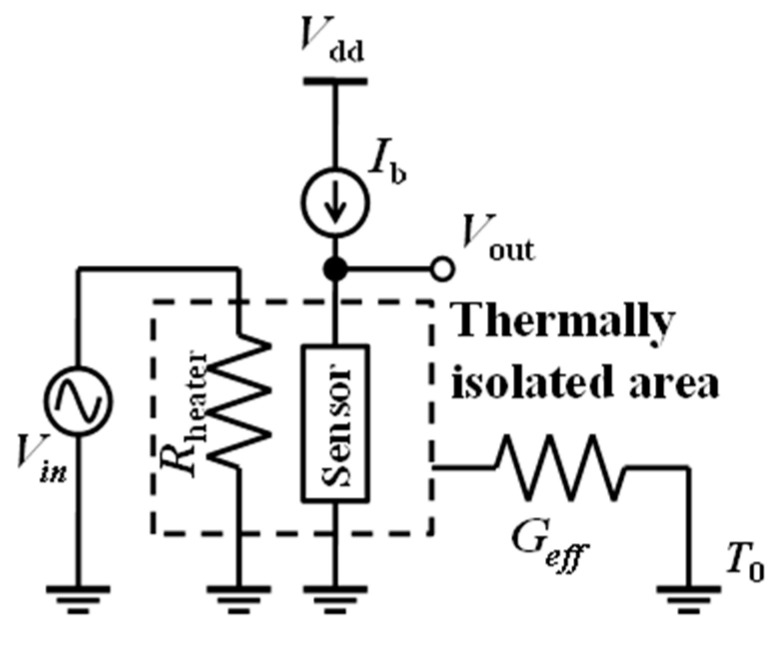
Equivalent circuit for responsivity (*R*_v_) estimation.

Figure 2 represents the electrical circuit for *R_v_* estimation. *R_v_* is the measure of output voltage at the temperature sensor for the corresponding applied sinusoidal input power (*P_in_*) at the heater under constant *I_b_* [46]. The structure and dimensions of MOSFET and diode bolometers are assumed to be the same, a similar measnuremental technique and modeling was used to calculate *R_v_*. The temperature dependence of output voltage (Δ*V_out_/*Δ*T*) was measured using a low-temperature prober Nagase Techno-Engineering Grail 21-205-6-LV-R and the Semiconductor Parameter analyzer Agilent 4156C on the fabricated devices. However, for diode bolometers the conversion has to be done on the measured results due to the variations in the device dimension. The conversion on measurement results were performed by considering the proportionality of the current to the aspect ratio (*W*/*L*). Temperature dependence of the heater input power (Δ*T/*Δ*P_in_*) was determined by implementing analytical modeling [47]. The parameters required for analytical modeling were extracted from the measured electrical characteristics. The measured and calculated electrical and thermal parameters are shown in Table 2. Parameters used in the analytical model are electrical resistance (*r_e_*), thermal resistance (*r_t_*), and TCR (*α*) of the polysilicon heater stacked with an SiO_2_ insulation layer, and thermal conductance of the source/drain or anode/cathode lead (*C*_c_).

The heater resistance was obtained from the current-voltage characteristics at room temperature. Thermal resistance can be evaluated from the slope of linear relationship between electrical resistance and square of the current. With *TCR* obtained from the measurement at different temperatures, thermal resistance can be extracted explicitly [48]. If there is a branch of thermal conduction [47], such as source/drain leads, the thermal conductance *C*_c_ can be obtained as a fitting parameter to reproduce the results from the samples with different dimensions. Reported values of specific heat, 1.6 × 10^6^ J/Km^3^ [49] for SiO_2_ and 1.66 × 10^6^ J/Km^3^ [50] for Si, were used to estimate the thermal capacitance and the response time (*τ*) of the bolometers.

Finally, *R*_v_ is evaluated from the temperature dependence of output voltage and input power as *R_v_* = (Δ*V_out_/*Δ*T*) × (Δ*T/*Δ*P_in_*). The temperature rise of FET bolometer is observed as a change in the gate threshold voltage (Δ*V*_gth_/Δ*T*). The amplification factors (*g_m_ R_o_*) are then multiplied with (Δ*V_gth_/*Δ*T*) to get the final output response, where *g*_m_ is the transconductance calculated from the drain current-gate voltage characteristics at 10 µA, and *R*_o_ is the output resistance obtained from the drain current-drain voltage characteristics at the drain voltage of 1 V. Similarly, in the diode bolometers, the change in the forward bias voltage (Δ*V_f_/*Δ*T*) was measured as a function of temperature.

**Figure 3:**
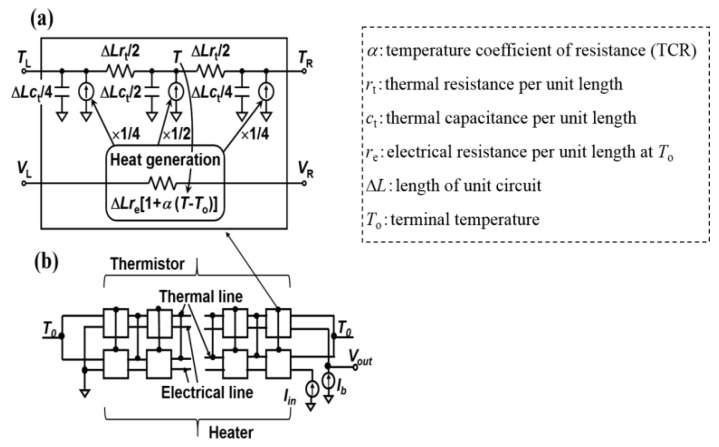
(**a**) Unit and (**b**) entire circuits of resistive bolometers for electrothermal simulation [51].

Electrothermal simulation was carried out using the SPICE circuit simulator to evaluate the *R_v_* of resistive bolometers [51] due to the lack of the fabricated device. Figure 3a represents a unit circuit of heater/thermistor with length Δ*L*, TCR (*α*), *r_e_*, *r_t_*, and *c*_t_. The entire circuit is depicted in Figure 3b in which the heater/sensor is stacked parallel. A bias current of 10 µA is applied to the temperature sensor. Temperature sensor parameters were extracted from the electrical measurements performed on the fabricated structure. Conversion from the fabricated device to the assumed device (length = 15 µm and width = 0.6 µm) was done by assuming the identical resistivity between them. The transient analysis was performed with a sinusoidal input under the CC bias of 10 µA in the SPICE circuit simulator for converting the temperature amplitude to the temperature sensor output voltage.

### 3.2. Estimation Procedure of Noise Equivalent Power (NEP) and Thermal Response Time (τ)

**Figure 4:**
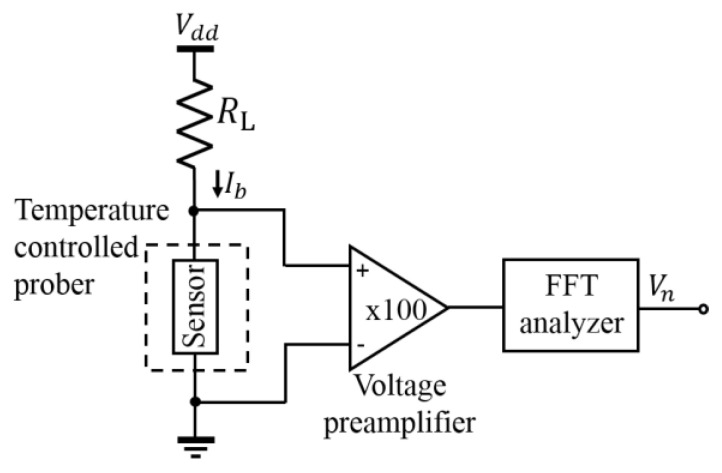
Circuit for noise measurement.

An important performance metric of thermal detectors is its NEP, which is defined as the input power that gives a signal-to-noise ratio of one for the output noise per unit bandwidth. Figure 4 represents the measurement setup for estimating voltage noise. The device being tested is mounted in the Nagase Techno-Engineering Grail 21-205-6-LV-R room temperature prober. The Agilent 35670A FFT dynamic signal analyzer has been used to measure the power spectral density of output voltage noise over a frequency range of 1 to 100 kHz together with the DL Instruments model 1201 low-noise voltage preamplifier with a gain of 100 and input-referred noise of less than 15 nV/Hz^1/2^ at 10 Hz. The input from the bolometer and the output of the amplifier were AC coupled to prevent overdriving the input of the spectrum analyzer due to the DC offset induced by *I*_b_.

For the estimation of *R_v_* we considered the CC load instead of the resistor load. However, for the noise measurement, the metal-film resistor *R*_L_ is connected as the load because the available CC load is noisy. The constant bias current of 10 µA was maintained by assuming the same current density for both fabricated and assumed bolometers. Since the dimensions of the measured and assumed bolometers are different and the loads are different, the output noise voltages of the measured bolometers are converted by the procedures explained in Appendix A. NEP is calculated from the estimated voltage noise (*V_n_*) at 10 Hz divided by *R*_v_. NEP in terms of W/Hz^1/2^ is expressed as:

(1) $$NEP=\frac{V_{n}}{R_{v}}$$

The bolometer response time or thermal time constant (*τ*) is generally expressed as the heat capacity (*C*) divided by thermal conductance (*G*). The measured cutoff frequency (*f*_c_) can be directly related to the *τ*. The *R_v_* of n-channel MOSFET was measured from the identical test structures by applying an amplitude-modulated input signal with the carrier frequency of 5 MHz and various modulation frequency (*f_m_*) to the heater to realize the flat response of the measurement system with respect to *f_m_* up to 100 kHz. The fundamental or second-harmonic output voltage (*f_m_* or 2*f_m_*) was recorded using a lock-in amplifier because the temperature rise is proportional to the square of the temporal amplitude of the modulated signal. The *τ* of the p-channel MOSFET and diode bolometers was obtained only by the compensation of the electrothermal feedback effect, which will be discussed later, since MOSFET and diode bolometers have the same structure except for the doping conditions. An electro thermal simulation based on the extracted parameters was carried out to evaluate the *f*_c_ of resistive bolometers.

## 4. Results and Discussion

As explained in Section 3, all the characterization was performed under the CC bias of 10 µA. The temperature rise in the bolometer due to the bias current crucially affects the performance metrics such as *R_v_* and *τ* due to the Joule self-heating through the positive TCR. According to [20], the effective thermal conductance *G_e_* modified by this electrothermal feedback (ETF) effect can be expressed for resistive bolometers with a constant-current load as:

*G_e_* = *G_o_*{1 − (*T*_1_ − *T_o_*)*α*}, (2)

where *T*_1_ and *T*_o_ are the bolometer and ambient temperatures, respectively. For a positive TCR (*α*), as in the case of metallic resistors, the positive feedback results in the smaller *G*_e_ and enhanced responsivity *R*_v_. The second term (*T_1_*–*T_o_*)*α* shows the extent of the effect.

As for the diode and MOSFET bolometers, effective TCR (*α*) can be defined respectively as:

*α* = (Δ*V_f_*/Δ*T*)/*V_f_* (3)

And

*α* = (Δ*V_gth_*/Δ*T*) × (*g_m_ R_o_*)/*V_d_*, (4)

where *V_f_* and *V*_d_ are the forward bias (anode) and drain voltages during operation, respectively.

Table 3 summarizes the extent of the ETF effect. Since the threshold voltage *V_gth_* of MOSFET and *V*_f_ of diode has negative temperature coefficients, the negative feedback, i.e., reduction of the *R*_v_ and increase in the *τ*, arises. It can be seen that the n- and p-channel MOSFET bolometers are affected appreciably, and others are not. Note that this effect is automatically included in the electrothermal simulation for resistive bolometers, but the effect on MOSFET and diode bolometers is compensated after the estimation of the *R*_v_.

**Figure 5:**
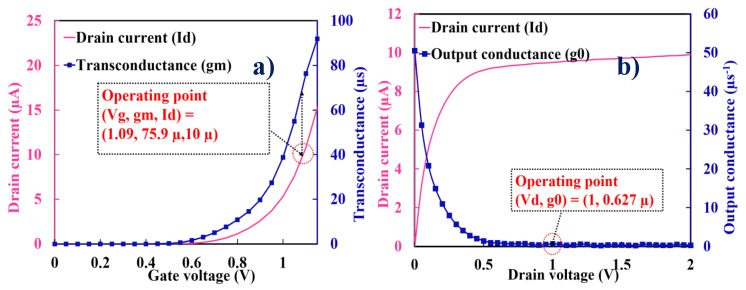
(**a**) Extraction of the transconductance (gm) from drain current vs. gate voltage characteristics; (**b**) extraction of the output conductance (g_0_) from drain current vs. drain voltage characteristics. As an example, characteristics of the n-channel MOSFET with *L* = 1 μm and *W* = 5 μm are shown. *V_d_* = 1.00 V for (**a**), and *V_g_* = 1.09 V for (**b**).

**Figure 6:**
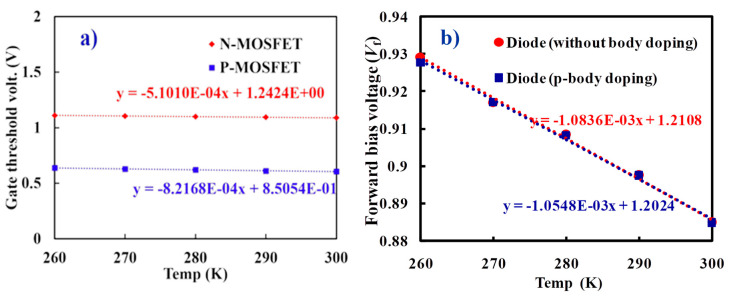
(**a**) Temperature dependence of the gate threshold voltage (MOSFET bolometer), and (**b**) temperature dependence of the forward bias voltage (diode bolometer).

**Figure 7:**
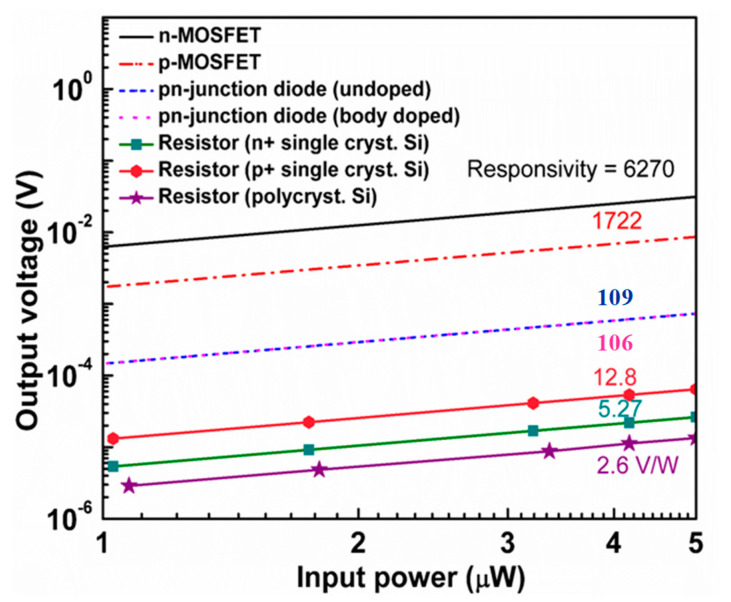
Estimated output voltage response of various bolometers for the heater input power. The ETF effect is not included in the lines for MOSFET and diode bolometers.

The *R*_v_ of MOSFET and diode bolometers before compensation of the ETF effect is estimated from the electrical measurement and analytical modeling. The gate threshold voltage shift and forward bias voltage shift take place as a result of the applied heater input power to MOSFET and diode bolometers, respectively. In the present MOSFET and diode bolometers, the input signal is applied to the heater (gate) and the resultant output signal is measured at the drain/anode under a constant bias current of 10 µA. Temperature dependence of the heater input power (Δ*T/*Δ*P_in_* = 1.43 × 10^5^ K/W) was evaluated by implementing analytical modeling. Since the common heater is assumed, the same value is used for the diode bolometer. Figure 5a,b represents the *I*_d_-*V*_g_ and *I*_d_-*V*_d_ characteristics of the MOSFET bolometer, respectively. Transconductance (*g_m_*) and output conductance (*g*_o_) required to calculate the amplification factor of MOSFET bolometers are extracted at 10 µA of the drain current (or bias current) and at 1 V of the drain voltage, respectively. The resultant amplification factors are 122 and 20.8 for the n and p-channel MOSFET, respectively. Temperature dependence of the gate threshold voltage (Δ*V_gth_/*Δ*T*) and forward bias voltage (Δ*V_f_/*Δ*T*) of MOSFET and diode bolometers are shown in Figure 6a,b, respectively. The Δ*T/*Δ*P_in_* and Δ*V_gth_/*Δ*T* together with the amplification factor gives the *R_v_* of MOSFET bolometer. Similarly, the Δ*T/*Δ*P_in_* and Δ*V_f_/*Δ*T* was used to compute the *R_v_* of diode bolometer. The *R_v_* of resistive bolometers were simulated based on the electrothermal circuit. Figure 7 presents the comparison of *R_v_* for the studied bolometers. The highest *R*_v_ was attained by the n-channel MOSFET bolometer, which is three orders of magnitude higher than those of the resistive bolometers.

The NEPs limited by the temperature fluctuation noise (thermal Johnson-Nyquist noise), and background fluctuation noise (photon noise) can be estimated as follows [20]:

*NEP_TF_* = (4*k_B_T*^2^*G*)^1/2^, (5)

And

*NEP_BF_* = (16*A_e_σk_B_T*^5^)^1/2^, (6)

where *k*_B_, T, G, *A*_e_, and *σ* are the Boltzman constant, temperature, thermal conductance, effective area of detector, and Stefan-Boltzman constant, respectively. Since the Gs for the present MOSFET/diode bolometer, the n^+^/p^+^ single crystalline, and n^+^ poly crystalline resistive bolometers are 3.5, 5.5, and 4.3 µW/K, the NEP_TF_s at 300 K are 4.2, 5.2, and 4.6 pW/Hz^1/2^, respectively. If we assume the bolometers coupled with a half-wave dipole antenna [52] *A*_e_ ~ 0.13*λ*^2^, then NEP_BF_ is calculated to be 0.6 pW/Hz^1/2^ at 300 K for the 1-THz operation. Since these NEPs are not dominant in the present bolometers, we will focus on the noises of the temperature sensors.

**Figure 8:**
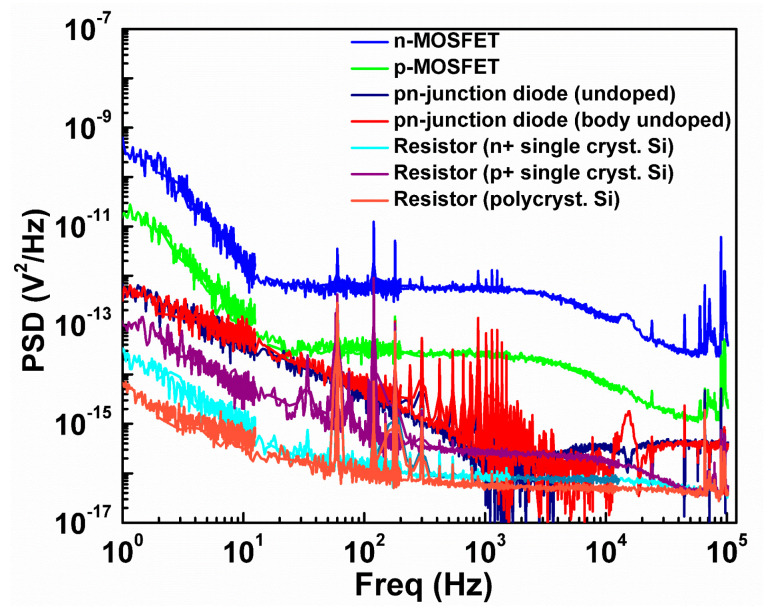
Voltage noise power spectral density (PSD) of investigated bolometers.

The voltage noise power spectral density (PSDs) of various bolometers are shown in Figure 8. Since the noise of MOSFETs shows a flicker or Lorenzian noise-like 1/*f ^n^* increase at low frequencies, noise levels at 10 Hz (near the corner frequency) are chosen to evaluate the NEP. This frequency is covered by the measurements with stop frequencies of 12.5 and 200 Hz, and the number of data points of 400. Although the equivalent noise band widths [53] are 47 and 750 mHz, respectively, the same PSDs are obtained at 10 Hz, indicating that the NEPs are not affected by the 1/*f* ^n^ behavior as long as the band width for the noise measurement is narrow. Spikes in the noise spectrum are due to the environmental noise. The baseline white noise is the thermal noise for MOSFETs and resistive thermistors, and shot noise for diodes. In the low-frequency region, the excess 1/*f* ^n^ noise is superimposed. The roll-off at a high frequency is caused by the limited band width of the measurement system.

Table 4 summarizes *R_v_*, NEP, and τ, in which the ETF effect is included. The noise equivalent temperature difference (NETD) is also calculated for the ease of comparison with other temperature sensors. Compared to the values in Figure 7, the *R_v_* of MOSFET bolometers, especially the n-channel MOSFET, gets degraded due to the ETF effect. Nevertheless, the n-channel MOSFET bolometer, which exhibits six times higher voltage gain compared to the p-channel one, shows the largest *R*_v_ of 5.16 kV/W due to the large amplifying function. The p-channel MOSFET bolometer attains the smallest NEP as the result of the superior trade-off between *R_v_* and noise. This can be explained by its low input-referred noise related to the buried-channel operation as a result of n-type channel doping with the n^+^ polysilicon gate. The resistive bolometers show that the noise voltage is smaller than those of other bolometers, but the NEP is worse due to the poor *R*_v_. Resistive bolometers with the n^+^/p^+^ single crystalline silicon thermistor have the smallest *τ* of 2.34 µs. Generally, the *τ*s obtained in this study are short, promising the operation band width of more than several kHz. For imaging applications, in which the ordinary frame rate is ~60 fps, there is a room for the improvement of *R_v_* by mitigating the response speed. The NETD as small as 23.1 µK/Hz^1/2^ is attained by the p-channel MOSFET, which results in the resolution figure-of-merit of 5.3 fJ·K^2^ comparable to 2.3 fJ·K^2^ by the state-of-the-art temperature sensor [54].

## 5. Conclusions

In this work, the main intension is to evaluate the performance of different Si-based temperature sensors integrated in antenna-coupled bolometers. To the best of our knowledge, this is the first report to make a fair comparison among different sensors under the common constraints of the silicon-on-insulator (SOI) CMOS design rule. The performances in terms of *R_v_*, NEP, and τ were evaluated for various temperature sensors such as MOSFETs (n- and p-channel), pn-junction diodes (with and without body doping), and resistors with different materials (n^+^ and p^+^ single-crystal Si, and n^+^ polycrystalline Si) for a 1-THz antenna-coupled bolometer. The ETF effect due to the bias current affected the performance of MOSFET bolometers appreciably. Even though, the n-channel MOSFET showed the largest *R_v_* of 5.16 kV/W. The high *R_v_* was mainly due to the signal amplifying function of the MOSFET. In addition, as the general characteristics of thin (~100 nm) SOI material, the reduced thermal conductance due to the enhanced phonon scattering could also contribute to the larger temperature rise, higher *R_v_*, and smaller NEP [48,55]. As the result of better compatibility between low noise and high *R_v_*, the p-channel MOSFET bolometer showed the smallest NEP of 170 pW/Hz^1/2^ at 10 Hz. Currently, the contribution of the thermal Johnson-Nyquist noise and the photon noise is not dominant, but would become an issue if we consider the passive imaging, in which NEP less than a few pW/Hz^1/2^ is required. The smallest thermal response time *τ* of 2.34 µs was attained by the n^+^/p^+^ resistive bolometers, whereas the n-channel MOSFET has the *τ* of 13.8 µs. For imaging applications, still there is a room for improvement in *R*_v_ by sacrificing the *τ* to the level of ordinary video frame rate. Considering the overall performance, i.e., high *R_v_* (high output voltage), moderate NEP, and *τ*, it can be concluded that the n-channel MOSFET at the center of an antenna can be a promising detector for the electromagnetic waves around 1 THz, which is useful for transparent imaging and material identification.

## Figures and Tables

**Table 1:** Assumed device parameters and dimensions.

Parameter	Fabricated Device Dimension (µm)	Assumed Device Dimension (µm)
Heater length of metal-oxide-semiconductor field-effect transistor (MOSFET) and diode	15	15
Heater width (gate length) of MOSFET and diode	1	1
Channel width of MOSFET	5	5
Width of pn-junction diode	50	5
Thermistor length of resistive bolometer	100	15
Thermistor width of resistive bolometer	1	0.6
Heater length of resistive bolometer	100	15
Heater width of resistive bolometer	1	1
Antenna width (W_ant_)	×	5.2

× The half-wave gold dipole antenna was not fabricated.

**Table 2:** Measured and calculated electrical/thermal parameters. *r_e_* and *r_t_* are the par unit length assuming thermistor and heater dimensions shown in Table 1.

Material	Measured Electrical and Thermal Parameters			Calculated Electrical and Thermal Parameters		
	Electrical Resistance (Ω)	Thermal Conductivity *k* (W/mK)	Temperature Coefficient of Resistance (TCR) (K^−1^)	Electrical Resistance *r_e_* (Ω/m)	Thermal Resistance *r_t_* (K/Wm)	Thermal Capacitance (J/Km)
Polysilicon (Heater) // SiO_2_	8.36 × 10^2^	21.6 (poly Si) 1.38 (SiO_2_)	1.11 × 10^−3^	5.57 × 10^7^	2.66 × 10^11^	1.31 × 10^−6^
n^+^ single crystalline silicon	2.63 × 10^3^	53.2	1.57 × 10^−3^	1.75 × 10^8^	3.13 × 10^11^	9.96 × 10^−5^
p^+^ single crystalline silicon	1.02 × 10^4^	53.2	9.83 × 10^−4^	6.83 × 10^8^	3.13 × 10^11^	9.96 × 10^−5^
Polysilicon (Thermistor)	1.44 × 10^3^	21.6	1.15 × 10^−3^	9.62 × 10^7^	5.92 × 10^11^	1.29 × 10^−7^

**Table 3:** Extent of the electrothermal feedback (ETF) effect. The temperature coefficient of resistances (TCRs) for MOSFETs and diodes are effective values.

Bolometers	*T*_1_−*T*_o_ (K)	TCR (K^−1^)	(*T*_1_−*T*_o_)*α* (%)
N-channel MOSFET	2.86	−6.18 × 10^−^^2^	−17.6
P-channel MOSFET	2.86	−1.71 × 10^−^^2^	−4.9
Diode (without body doping)	2.53	−1.22 × 10^−3^	−0.31
Diode (with p-body doping)	2.53	−1.19 × 10^−3^	−0.30
Resistive (n^+^ single crystalline Si)	4.74 × 10^−^^2^	1.57 × 10^−3^	~0
Resistive (p^+^ single crystalline Si)	1.85 × 10^−^^1^	9.83 × 10^−4^	~0
Resistive (polycrystalline Si)	3.32 × 10^−^^2^	1.15 × 10^−3^	~0

**Table 4:** Performance comparison among the studied bolometers. The ETF effect is considered in *R*_v_, noise equivalent power (NEP), and *τ*, but is not in the noise equivalent temperature difference (NETD) based on the assumption of isothermal measurement.

Bolometers	Voltage Noise at 10 Hz (V/Hz^1/2^)	Responsivity R*_v_* (V/W)	NEP (W/Hz^1/2^)	Response Time *τ* (μs)	NETD (K/Hz^1/2^)
N-channel MOSFET	1.27 × 10^−6^	5.16 k	2.45 × 10^−10^	13.8	2.88 × 10^−5^
P-channel MOSFET	2.79 × 10^−7^	1.64 k	1.70 × 10^−10^	15.9	2.31 × 10^−5^
Diode (without body doping)	2.08 × 10^−7^	109	1.99 × 10^−9^	16.7	2.83 × 10^−4^
Diode (with p- doping)	2.27 × 10^−7^	106	2.15 × 10^−9^	16.7	3.05 × 10^−4^
Resistive (n^+^ single-Si)	2.70 × 10^−8^	5.27	5.12 × 10^−9^	2.34	9.23 × 10^−4^
Resistive (p^+^ single-Si)	7.72 × 10^−8^	12.8	6.01 × 10^−9^	2.34	1.07 × 10^−3^
Resistive (n^+^ poly-Si)	1.78 × 10^−8^	2.69	6.59 × 10^−9^	3.07	1.52 × 10^−3^

**Figure A1:**
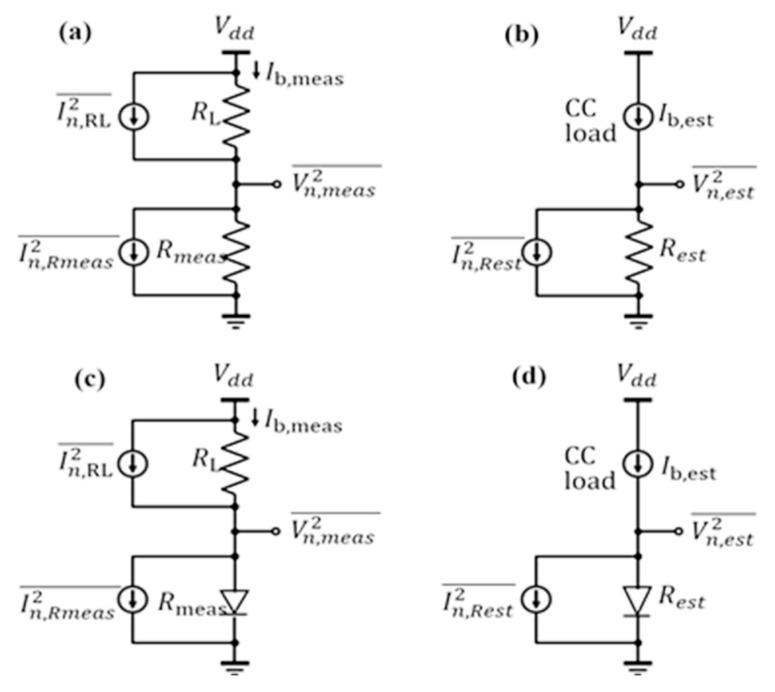
Noise modeling for the measured (fabricated) and estimated (assumed) bolometers. (**a**) Measured noise for the available resistive bolometer with a resistive load, (**b**) estimated noise for the designed resistive bolometer with a CC load, (**c**) measured noise for the available diode bolometer with a resistive load, (**d**) estimated noise for the designed diode bolometer with a CC load.

## Appendix A. Conversion Procedure of the Output Noise Voltages

MOSFET Bolometer

For the FET bolometer, an input referred noise is estimated from the measured noise $\bar{\left(V_{n,meas}^{2}\right)}$ divided by the voltage gain $\left(A_{v,meas}\right)$ with load resistance *R*_L_ and can be expressed as:

(A1) $$\bar{V_{in.meas}^{2}}=\frac{\bar{V_{n,meas}^{2}}}{{\left(A_{v,meas}\right)}^{2}}$$

The voltage gain of the FET including the load resistor $R_{L}$ is expressed as:

(A2) $$A_{v,meas}=g_{m}.(R_{meas}||R_{L})$$

The CC load voltage noise $\left(\bar{V_{n,est}^{2}}\right)$ is then calculated by multiplying the input referred noise $\bar{(V_{in.meas}^{2}})$ with the voltage gain $\left(A_{v,est}\right)$ without *R*_L_ which can be written as:

(A3) $$\bar{V_{n,est}^{2}}=\bar{V_{in.meas}^{2}}.{\left(A_{v,est}\right)}^{2}$$

(A4) $$A_{v,est}=g_{m}.R_{meas}$$

Diode and Resistive Bolometer

The noise voltage of the assumed diode and resistive bolometer at the CC load was estimated based on the circuit diagram shown in Figure A1.

For the pn-junction diode bolometer, noise has been estimated by considering both shot noise and thermal noise. The effect of *R*_L_ was eliminated by subtracting the theoretical thermal noise from the measured noise, which can be expressed as:

(A5) $$\bar{V_{n,meas\left(without\;RL\right)}^{2}}=\bar{V_{n,meas\left(with\;RL\right)}^{2}}-\bar{V_{n.RL}^{2}}$$

Since the shot noise originates from the electric charge, the measured voltage noise was converted to the current noise for further conversion. The assumed diode bolometer current noise $\left(\bar{I_{n,est}^{2}}\right)$ can be expressed as:

(A6) $$\bar{I_{n,est}^{2}}=\left\{\frac{\frac{4.k.T}{R_{meas}}+2qI_{b,meas}}{\frac{4.k.T}{R_{est}}+2qI_{b,est}}\right\}.\bar{I_{n,meas}^{2}}$$

The current noise of the diode bolometer is the converted to voltage noise by:

(A7) $$\bar{V_{n,est}^{2}}=\bar{I_{n,est}^{2}}.\;{\left(R_{est}\right)}^{2}$$

$I_{b,meas}$ and $I_{b,est}$ are the bias currents of fabricated and assumed devices, respectively. Similarly, $R_{meas}$ and $R_{est}$ are resistances of fabricated and assumed diode bolometers.

For the resistive bolometer in which the thermal noise is of major concern the conversion equation can be written as:

(A8) $$\bar{V_{n,est}^{2}}=\bar{V_{n,meas}^{2}}.\left\{\frac{R_{est}}{R_{meas}||R_{L}}\right\}$$

where $R_{meas}$ and $R_{est}$ are the resistances of fabricated and assumed devices, respectively.

## References

[1] Kukutsu N., Kado Y. (2009). Overview of millimeter and terahertz wave application research. NTT Tech. Rev..

[2] Zhu B., Chen Y., Deng K., Hu W., Yao Z.S. (2009). Terahertz Science and Technology and Applications. PIERS Proc..

[3] Rogalski A. (1997). Infrared thermal detectors versus photon detectors: I. Pixel performance. Mater. Sci. Mater. Prop. Infrared Optoelectron..

[4] Li H., Wan W.J., Tan Z.Y., Fu Z.L., Wang H.X., Zhou T., Li Z.P., Wang C., Guo X.G., Cao J.C. (2017). 6.2-GHz modulated terahertz light detection using fast terahertz quantum well photodetectors. Sci. Rep..

[5] Okamoto T., Fujimura N., Crespi L., Kodera T., Kawano Y. (2019). Terahertz detection with an antenna-coupled highly-doped silicon quantum dot. Sci. Rep..

[6] Palaferri D., Todorov Y., Chen Y.N., Madeo J., Vasanelli A., Li L.H., Davies A.G., Linfield E.H., Sirtori C. (2015). Patch antenna terahertz photodetectors. Appl. Phys. Lett..

[7] Zhang Y., Hosono S., Nagai N., Song S.-H., Hirakawa K. (2019). Fast and sensitive bolometric terahertz detection at room temperature through thermomechanical transduction. J. Appl. Phys..

[8] Kreisler A.J.M. (1986). Submillimeter Wave Applications of Submicron Schottky Diodes. Far-Infrared Sci. Technol..

[9] Crowe T.W., Mattauch R.J., Weikle R.M., Bhapkar U.V. (1995). Terahertz Gaas Devices and Circuits for Heterodyne Receiver Applications. Int. J. High. Speed Electron. Syst..

[10] Han R., Zhang Y., Kim Y., Kim D.Y., Shichijo H., Afshari E., Kenneth K.O. (2013). Active Terahertz Imaging Using Schottky Diodes in CMOS: Array and 860-GHz Pixel. IEEE J. Solid-State Circuits.

[11] Knap W., Teppe F., Meziani Y., Dyakonova N., Lusakowski J., Boeuf F., Skotnicki T., Maude D., Rumyantsev S., Shur M.S. (2004). Plasma wave detection of sub-terahertz and terahertz radiation by silicon field-effect transistors. Appl. Phys. Lett..

[12] Tauk R., Teppe F., Boubanga S., Coquillat D., Knap W., Meziani Y.M., Gallon C., Boeuf F., Skotnicki T., Fenouillet-Beranger C. (2006). Plasma wave detection of terahertz radiation by silicon field effects transistors: Responsivity and noise equivalent power. Appl. Phys. Lett..

[13] Golenkov A.G., Sizov F.F. (2016). Performance limits of terahertz zero biased rectifying detectors for direct detection. Semicond. Phys. Quantum Electron. Optoelectron..

[14] Földesy P., Gergelyi D., Füzy C., Károlyi G. Test and configuration architecture of a sub-THz CMOS detector array. Proceedings of the 15th International Symposium on Design and Diagnostics of Electronic Circuits & Systems (DDECS).

[15] Bauer M., Boppel S., Lisauskas A., Krozer V., Roskos H.G. Real-time CMOS terahertz camera employing plane-to-plane imaging with a focal-plane array of field-effect transistors. Proceedings of the 38th International Conference on Infrared, Millimeter, and Terahertz Waves (IRMMW-THz).

[16] Perenzoni D., Perenzoni M., Gonzo L., Capobianco A.D., Sacchetto F. (2010). Analysis and design of a CMOS-based terahertz sensor and readout. Opt. Sens. Detect..

[17] Schuster F., Coquillat D., Videlier H., Sakowicz M., Teppe F., Dussopt L., Giffard B., Skotnicki T., Knap W. (2011). Broadband terahertz imaging with highly sensitive silicon CMOS detectors. Opt. Express.

[18] Lisauskas A., Boppel S., Roskos H.G., Matukas J., Palenskis V., Minkevičius L., Valušis G., Haring Bolivar P. Terahertz responsivity enhancement and low-frequency noise study in silicon CMOS detectors using a drain current bias. Proceedings of the 21st International Conference on Noise and Fluctuations.

[19] Nemirovsky Y., Svetlitza A., Brouk I., Stolyarova S. (2013). Nanometric CMOS-SOI-NEMS transistor for uncooled THz sensing. IEEE Trans. Electron. Devices.

[20] Kruse P.W. (2001). Uncooled Thermal Imaging. Arrays, Systems, and Applications.

[21] Garn L.E. (1984). Fundamental noise limits of thermal detectors. J. Appl. Phys..

[22] Neikirk D.P., Lam W.W., Rutledge D.B. (1984). Far-infrared microbolometer detectors. Int. J. Infrared Millim. Waves.

[23] Chen S., Ma H., Xiang S., Yi X. (2007). Fabrication and performance of microbolometer arrays based on nanostructured vanadium oxide thin films. Smart Mater. Struct..

[24] Chen C., Yi X., Zhao X., Xiong B. (2001). Characterizations of VO2-based uncooled microbolometer linear array. Sensors Actuators A Phys..

[25] Chen C., Yi X., Zhang J., Zhao X. (2001). Linear uncooled microbolometer array based on VOx thin films. Infrared Phys. Technol..

[26] Wang H., Yi X., Huang G., Xiao J., Li X., Chen S. (2004). IR microbolometer with self-supporting structure operating at room temperature. Infrared Phys. Technol..

[27] Bhan R.K., Saxena R.S., Jalwania C.R., Lomash S.K. (2009). Uncooled infrared microbolometer arrays and their characterisation techniques. Def. Sci. J..

[28] Lee H.K., Yoon J.B., Yoon E., Ju S.B., Yong Y.J., Lee W., Kim S.G. (1999). A high fill-factor infrared bolometer using micromachinedmultilevelelectrothermal structures. IEEE Trans. Electron. Devices.

[29] Banerjee A., Satoh H., Sharma Y., Hiromoto N., Inokawa H. (2018). Characterization of platinum and titanium thermistors for terahertz antenna-coupled bolometer applications. Sens. Actuators A Phys..

[30] Tezcan D.S., Eminoglu S., Akin T. (2003). A low-cost uncooled infrared microbolometer detector in standard CMOS technology. IEEE Trans. Electron. Devices.

[31] Niklaus F., Kälvesten E., Stemme G. (2001). Wafer-level membrane transfer bonding of polycrystalline silicon bolometers for use in infrared focal plane arrays. J. Micromech. Microeng..

[32] Niklaus F., Pejnefors J., Dainese M., Haggblad M., Hellstrom P.E., Wallgren U.J., Stemme G. (2004). Characterization of transfer-bonded silicon bolometer arrays. Proc. SPIE.

[33] Simoens F., Meilhan J. (2014). Terahertz real-time imaging uncooled array based on antenna- and cavity-coupled bolometers. Philos. Trans. R. Soc. A Math. Phys. Eng. Sci..

[34] Ueno M., Kosasayama Y., Sugino T., Nakaki Y., Fujii Y., Inoue H., Kama K., Seto T., Takeda M., Kimata M. (2005). 640 × 480 pixel uncooled infrared FPA with SOI diode detectors. Proc. SPIE.

[35] Ishikawa T., Ueno M., Nakaki Y., Endo K., Ohta Y., Nakanishi J., Kosasayama Y., Yagi H., Sone T., Kimata M. (2000). Performance of 320 × 240 uncooled IRFPA with SOI diode detectors. Proc. SPIE.

[36] Kimata M., Ueno M., Takeda M., Seto T. (2006). SOI diode uncooled infrared focal plane arrays. Proc. SPIE.

[37] Olgun Z., Akar O., Kulah H., Akin T. An integrated thermopile structure with high responsivity using any standard CMOS process. Proceedings of the International Solid State Sensors and Actuators Conference (Transducers’97).

[38] Gitelman L., Stolyarova S., Bar-Lev S., Gutman Z., Ochana Y., Nemirovsky Y. (2009). CMOS-SOI-MEMS transistor for uncooled IR imaging. IEEE Trans. Electron. Devices.

[39] Morf T., Klein B., Despont M., Drechsler U., Kull L., Corcos D., Elad D., Kaminski N., Pfeiffer U.R., Al Hadi R. (2014). Wide bandwidth room-temperature THz imaging array based on antenna-coupled MOSFET bolometer. Sensors Actuators A Phys..

[40] Hiromoto N., Tiwari A., Aoki M., Satoh H., Takeda M., Inokawa H. Room-temperature THz antenna-coupled microbolometer with a Joule-heating resistor at the center of a half-wave antenna. Proceedings of the 38th International Conference on Infrared, Millimeter, and Terahertz Waves (IRMMW-THz).

[41] Kruse P.W. (1995). A comparison of the limits to the performance of thermal and photon detector imaging arrays. Infrared Phys. Technol..

[42] Rogalski A. New trends in infrared and terahertz detectors. Proceedings of the Conference on Optoelectronic and Microelectronic Materials & Devices.

[43] Kominami M., Pozar D.M., Schaubert D.H. (1985). Dipole and slot elements and arrays on semi-infinite substrate. IEEE Trans. Antennas Propag..

[44] Banerjee A., Satoh H., Tiwari A., Apriono C., Rahardjo E.T., Hiromoto N., Inokawa H. (2017). Width dependence of platinum and titanium thermistor characteristics for application in room-temperature antenna-coupled terahertz microbolometer. Jpn. J. Appl. Phys..

[45] Tiwari A., Satoh H., Aoki M., Takeda M., Hiromoto N., Inokawa H. (2015). THz Antenna-Coupled Microbolometer with 0.1-µm-wide Titanium Thermistor. Intern. J. ChemTech Res..

[46] Datskos P.G., Lavrik N.V., Driggers R.G. (2003). Detector—figure of merits. Encyclopedia of Optical Engineering.

[47] Tiwari A., Satoh H., Aoki M., Takeda M., Hiromoto N., Inokawa H. (2015). Fabrication and analytical modeling of integrated heater and thermistor for antenna-coupled bolometers. Sens. Actuators A Phys..

[48] Liu W., Asheghi M. (2005). Thermal conduction in ultrathin pure and doped single-crystal silicon layers at high temperatures. J. Appl. Phys..

[49] van Herwaarde S. (1996). Physical principles of thermal sensors. Sens. Mater..

[50] National Astronomical Observatory of Japan (2015). Chronological Scientific Tables.

[51] Tiwari A., Satoh H., Aoki M., Takeda M., Hiromoto N., Inokawa H. (2013). Analysis of Microbolometer Characteristics for Antenna-Coupled THz Detectors. Asian, J. Chem..

[52] Kraus J.D., Marhefka R.J. (2002). Antennas for All Applications.

[53] Harris F.J. (1978). On the use of windows for harmonic analysis with the discrete Fourier transform. Proc. IEEE.

[54] Pan S., Luo Y., Shalmany S.H., Makinwa K.A.A. (2018). A Resistor-Based Temperature Sensor With a 0.13 pJ·K^2^ Resolution FoM. IEEE J. Solid-State Circuits.

[55] Boukai A.I., Bunimovich Y., Kheli J.T., Yu J.K., Goddard W.A., Heath J.R. (2008). Silicon nanowires as efficient thermoelectric materials. Nature.

